# Phytochemical Quantification and the In Vitro Acetylcholinesterase Inhibitory Activity of *Phellodendron chinense* and Its Components

**DOI:** 10.3390/molecules22060925

**Published:** 2017-06-02

**Authors:** Yu Jin Kim, Hye-Sun Lim, Yoonju Kim, Jun Lee, Bu-Yeo Kim, Soo-Jin Jeong

**Affiliations:** 1 Herbal Medicine Research Division, Korea Institute of Oriental Medicine, Daejeon 34054, Korea; jinjin0228@kiom.re.kr (Y.J.K.); qp1015@kiom.re.kr (H.-S.L.); yoonjukim@kiom.re.kr (Y.K.); junlee@kiom.re.kr (J.L.); buykim@kiom.re.kr (B.-Y.K.); 2 College of Pharmacy, Chungnam National University, Daejeon 34134, Korea; 3 Korean Medicine of Life Science, University of Science & Technology, Daejeon 34113, Korea

**Keywords:** *Phellodendron chinense*, phellodendrine, quantitative analysis, neuroprotection

## Abstract

The dried bark of *Phellodendron chinense* has been used as a traditional herbal medicine to remove damp heat, relieve consumptive fever, and cure dysentery and diarrhea. In the present study, we performed quantitative analyses of the two components of *P. chinense*, phellodendrine and berberine, using high-performance liquid chromatography. A 70% ethanol extract of *P. chinense* was prepared and the two components were separated on a C-18 analytical column using a gradient solvent system of acetonitrile and 0.1% (*v*/*v*) aqueous trifluoroacetic acid. The ultraviolet wavelength used for detection was 200 nm for phellodendrine and 226 nm for berberine. The analytical method established here showed high linearity (correlation coefficient, ≥0.9991). The amount of phellodendrine and berberine used was 22.255 ± 0.123 mg/g and 269.651 ± 1.257 mg/g, respectively. Moreover, we performed an in vitro acetylcholinesterase (AChE) activity assay and an amyloid-β aggregation test to examine the biological properties of phellodendrine and berberine as therapeutic drugs for Alzheimer’s disease. Phellodendrine and berberine inhibited AChE activity in a dose-dependent manner (IC_50_ = 36.51 and 0.44 μM, respectively). In contrast, neither phellodendrine nor berberine had an effect on amyloid-β aggregation. The *P. chinense* extract and phellodendrine, but not berberine, exhibited antioxidant activity by increasing radical scavenging activity. Moreover, *P. chinense* demonstrated a neuroprotective effect in hydrogen peroxide-treated HT22 hippocampal cells. Overall, our findings suggest that *P. chinense* has potential as an anti-Alzheimer’s agent via the suppression of the enzymatic activity of acetylcholinesterase and the stimulation of antioxidant activity.

## 1. Introduction

Alzheimer’s disease is a type of dementia that is associated with a loss of memory and other intellectual abilities that is sufficiently serious to interfere with daily life. Alzheimer’s disease is the most common form of dementia and accounts for about 60–70% of all dementia cases [1,2]. Risk factors for Alzheimer’s disease include aging and genetic problems, with several genes being involved [3]. Although Alzheimer’s disease is the seventh leading cause of death worldwide [4], no therapeutic method has been developed. Currently, acetylcholinesterase (AChE) inhibition and amyloid-β aggregation are considered as the two pivotal targets for developing anti-Alzheimer’s disease drug(s) [5,6].

*Phellodendron chinense* (*P. chinense*) is a medicinal plant in the genus *Phellodendron*, traditionally used to treat damp heat, relieve consumptive fever, and cure dysentery and diarrhea [7,8]. *P. chinense* has also been medicinally used to quench fire, counteract toxicity, and cure heat accumulation in the intestines and stomach. In addition, the potential of *P. chinense* was reported as an immune-modulator [9]. Thus, *P. chinense* is a major composition of various herbal formulae such as Hwanglyeonhaedok-tang and Dangguiyukhwang-tang. Of interest, recent scientific reports determined the biological activities of *P. chinense* and its active components, which include anticancer [10,11], anti-inflammation [12], antidiabetes (and its complications) [13], and antialgal [14] activities, as well as neuroprotection [15]. However, Xian et al. [15] described the neuroprotective effect of *P. chinense* using PC12 cells derived from pheochromocytoma of the rat adrenal medulla, and not neuronal cells. They did not examine whether the components of *P. chinense* have the same biological activity. In the present study, we investigated the inhibitory effect of *P. chinense* and its two standard components on the key biomarkers of Alzheimer’s disease and neuronal cell damage using HT22 murine hippocampal cells. We also performed a high-performance liquid chromatography (HPLC) analysis to obtain quantitative information regarding the two components of *P. chinense*.

## 2. Results

### 2.1. Optimization of HPLC Separation

**Figure 1:**
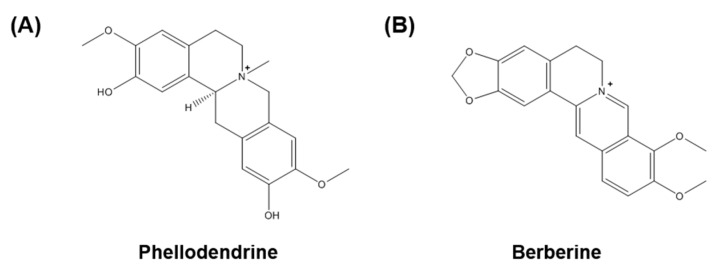
Chemical structures of the two marker compounds of *P. chinense*: (**A**) phellodendrine and (**B**) berberine.

**Figure 2:**
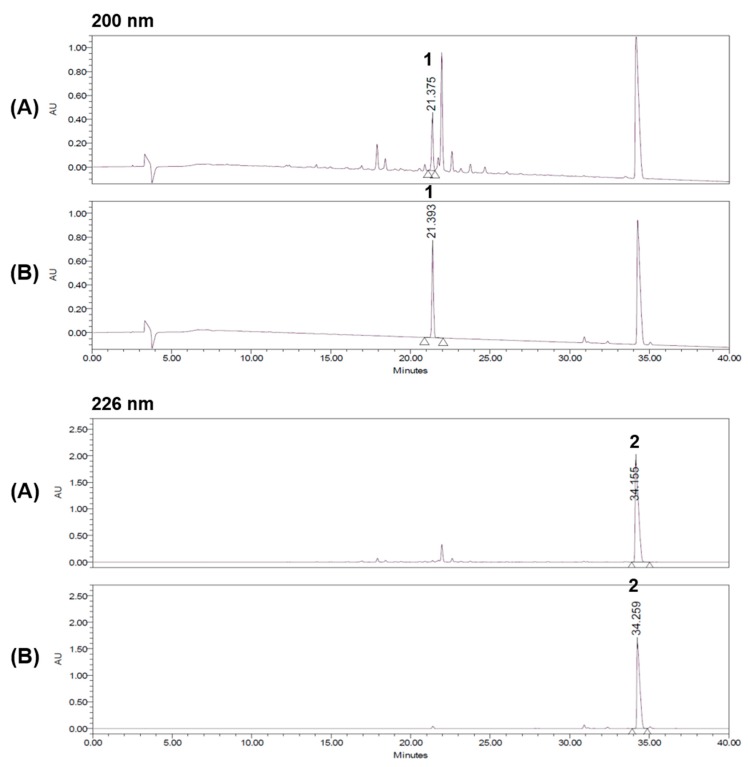
HPLC chromatograms of the 70% ethanol extract of the bark of *P. chinense* (**A**) and a standard mixture (**B**) at 200 nm and 226 nm. Phellodendrine (**1**) and berberine (**2**).

We used HPLC for the separation of the two standard components of the 70% ethanol extract of *P. chinense*. The established condition of the mobile phase is shown in Table 1. In the detector wavelength ranging from 190 to 400 nm, ultraviolet (UV) recordings were performed at a wavelength of 200 nm for phellodendrine (Figure 1A) and 226 nm for berberine (Figure 1B). Using this HPLC method, the two standard components were resolved within 35 min. The retention times of phellodendrine and berberine were 21.36 min and 34.14 min, respectively. The HPLC chromatograms of the 70% ethanol extract of *P. chinense* and the standard mixture are shown in Figure 2.

### 2.2. Linearity, Limit of Detection (LOD), and Limit of Quantitation (LOQ)

The linear relationships between the peak areas (*y*) and concentrations (*x*, μg/mL) of the components are expressed by the regression equations (*y* = a*x* + b) given in Table 2. The linearity of the analytical method established here was evaluated based on the correlation coefficient (*r*^2^) value of the calibration curves of each component. The calibration curves showed linearity, with *r*^2^ values ≥0.9991 over the concentration ranges of 12.5–200 μg/mL for phellodendrine and 62.5–1000 μg/mL for berberine. The LOD and LOQ for the two standard components, phellodendrine and berberine, were 0.301 and 1.555 μg/mL and 0.911 and 4.712 μg/mL, respectively.

### 2.3. Determination of the Two Standard Components of P. chinense

The HPLC analytical method established here was applied to the simultaneous quantification of the two components of *P. chinense*. The amount of the two standard components is shown in Table 3. Of the two components, berberine (269.651 mg/g) was the most abundant compared with phellodendrine (22.255 mg/g) in the bark of *P. chinense*.

### 2.4. Inhibitory Effects of the P. chinense Extract and Its Standard Components on AChE Activity

**Figure 3:**
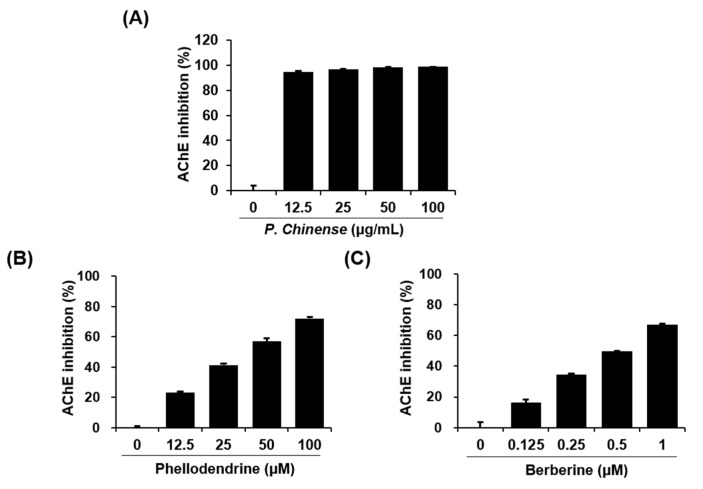
Effects of an ethanol extract of *P. chinense* and its components on acetylcholinesterase (AChE) activity. The AChE activity assay was performed using a modified Ellman’s colorimetric method. The enzymatic reaction was performed by incubating the mixture of AChE solution and various concentrations of the *P. chinense* extract (**A**); phellodendrine (**B**); and berberine (**C**) for 1 h at room temperature. The absorbance was measured at 412 nm using an Epoch microplate spectrophotometer (Bio-Tek Instruments, Winooski, VT, USA). Each value is presented as the mean ± SEM (*n* = 3).

To determine the biological activity of the *P. chinense* extract, its effects on the activation of AChE and the aggregation of amyloid-β, both of which are key events in Alzheimer’s disease, were assessed. The *P. chinense* extract dramatically inhibited AChE activity at 100 μg/mL. In contrast, no significant effect was observed on amyloid-β aggregation (Table 4). Inhibition of AChE activity by the *P. chinense* extract was further confirmed at lower concentrations, including 12.5, 25 or 50 μg/mL. As shown in Figure 3A, the *P. chinense* extract markedly decreased the activity of AChE, even at 12.5 μg/mL. Additional assays were performed to examine whether the standard components of *P. chinense* affect AChE activity. Both components increased the inhibition of AChE activity in a dose-dependent manner (Figure 3B,C). All experiments were carried out at ranges of the non-toxic concentrations based on results of the cytotoxicity assays against neuronal cells (data not shown).

### 2.5. Antioxidant Activity of the P. chinense Extract and Its Standard Components

**Figure 4:**
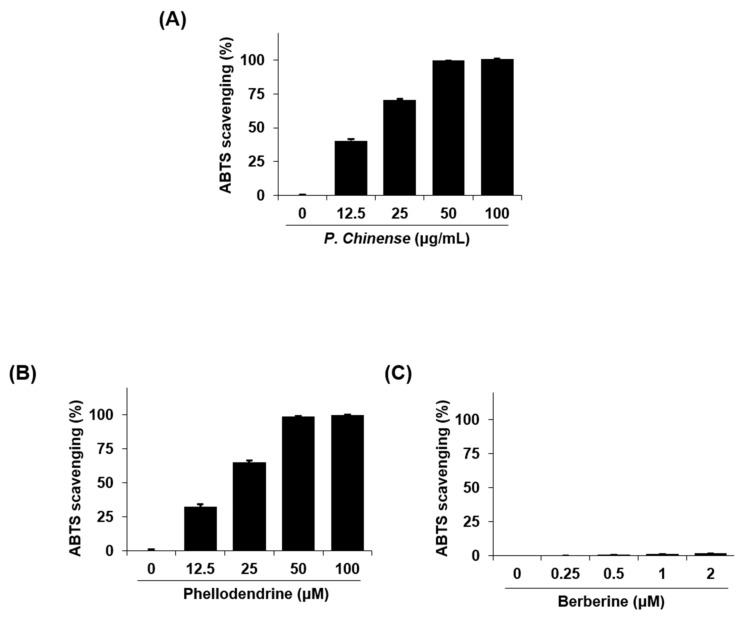
The 2,2′-azino-bis(3-ethylbenzothiazoline-6-sulphonic acid) (ABTS) radical scavenging activity of an ethanol extract of *P. chinense* and its components: (**A**) ethanol extract of *P. chinense*; (**B**) phellodendrine; and (**C**) berberine. ABTS scavenging activities are equal to the percentage inhibition of the ABTS radical. Each value is presented as the mean ± SEM (*n* = 3).

The antioxidant effect of the *P. chinense* extract was determined by measuring the 2,2′-azino-bis(3-ethylbenzothiazoline-6-sulphonic acid) (ABTS) radical scavenging activities. As shown in Figure 4A, the *P. chinense* extract increased the radical scavenging activity of ABTS in a dose-dependent manner. Of its two standard components, phellodendrine, but not berberine, enhanced the ABTS radical scavenging activity in a dose-dependent manner (Figure 4B,C).

### 2.6. Neuroprotective Effects of the P. chinense Extract in HT22 Neuronal Cells

**Figure 5:**
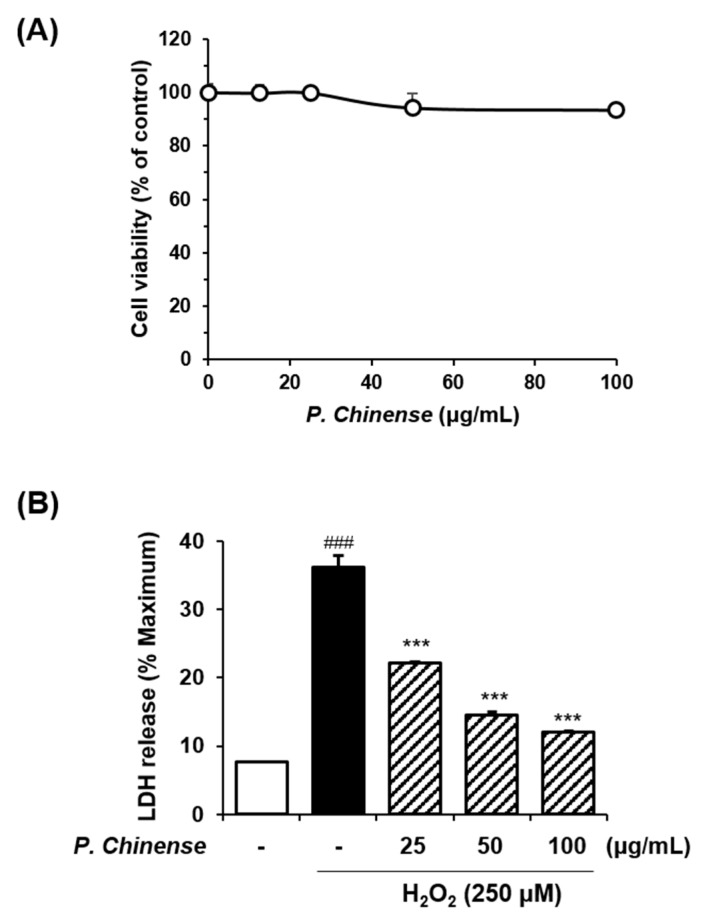
Neuroprotective effect of an ethanol extract of *P. chinense* in HT22 hippocampal cells. (**A**) Cells were seeded in 96-well plates and treated with various concentrations (0, 12.5, 25, 50 or 100 μg/mL) of *P. chinense* for 24 h. Cell viability was assessed using the Cell Counting Kit (CCK)-8 assay; (**B**) Neuroprotection was tested using the lactose dehydrogenase (LDH) release assay. As an indicator of cell disruption, LDH release from the cells was measured in supernatants and cell lysates. The results are expressed as the mean ± SEM of three independent experiments. ^###^ *p* < 0.001 vs. vehicle control cells; *** *p* < 0.001 vs. H_2_O_2_-treated cells.

We further confirmed the biological activity of the *P. chinense* extract on Alzheimer’s disease using HT22 murine hippocampal cells. A Cell Counting Kit (CCK) assay was performed to examine the viability of HT22 cells. Cells were treated with various concentrations of *P. chinense* for 24 h. Treatment with *P. chinense* extract had no significant effect on cell viability at a concentration ≤100 μg/mL (Figure 5A).

Subsequently, the release of lactose dehydrogenase (LDH) was measured to investigate whether the *P. chinense* extract has a protective effect in neuronal cells. HT22 cells were exposed to hydrogen peroxide (H_2_O_2_) with or without the *P. chinense* extract. H_2_O_2_ administration significantly increased LDH release compared with the untreated control. In contrast, the *P. chinense* extract significantly reduced the levels of LDH in H_2_O_2_-treated HT22 cells compared with H_2_O_2_ alone (Figure 5B).

## 3. Discussion

The traditional medicinal herb *P. chinense* has been used to reduce heat and fever and to release toxins in Asian countries, including Korea, China, and Japan [10]. Here, we demonstrated that an ethanol extract of *P. chinense* had a potent effect on Alzheimer’s disease-related in vitro bioevents, such as AChE activation, antioxidation, and the protection against neuronal cell damage. We also investigated the concentration of the chemical components of *P. chinense* and its biological activities regarding Alzheimer’s disease-related factors.

To develop a quality control for *P. chinense*, a quantitative analysis of its chemical constituents, including phellodendrine and berberine, was performed using HPLC-PDA. As a result, the contents of phellodendrine and berberine were determined to be 22.255 ± 0.123 mg/g and 269.651 ± 1.257 mg/g, respectively. Phellodendrine has been reported to have antioxidant and anti-inflammatory properties [16], and to act as an immunosuppressor [17,18]. Many studies have reported the biological and clinical efficacies of berberine from several different herbal plants, such as *Berberis vulgaris*, *Hydrastis canadensis*, *Xanthorhiza simplicissima*, *P. amurense*, and *Coptis chinensis*. Berberine is known to be a potential therapeutic agent for diabetes [19], hyperlipidemia [20], and cancers [21,22,23]. Recently, Kaufmann et al. reported the anti-Alzheimer’s disease effect of a combined treatment with berberine, coptisine, and palmatine, by targeting AChE activity [24]. However, those authors focused on the synergistic effect of the three alkaloids and evaluated only AChE activity as a biological marker of Alzheimer’s disease.

AChE is an enzyme that is present in the central nervous system and hydrolyzes acetylcholine and acetate, mainly functioning as a neurotransmitter. Currently, AChE inhibitors are the gold-standard treatment for Alzheimer’s disease [25]. For example, donepezil, rivastigmine, and galantamine are the major therapies approved by the US Food and Drug Administration (FDA) and the European Medicines Agency for the symptomatic treatment of Alzheimer’s disease [26]. However, these agents are only able to alleviate minor symptomatic problems and are not curative. They also cause serious side effects, such as bradycardia, hypotension, hypersecretion, bronchoconstriction, gastrointestinal tract hypermotility, and decreased intraocular pressure. Therefore, a new drug discovery for Alzheimer’s disease is required, with more attention to the AChE inhibition and less side effects. Many papers have reported the potential of herbal plants as AChE inhibitors to overcome the problems of synthetic drugs. For instance, Saleem et al. reported the significant inhibitory activity of *Jatropha gossypyfolia* leaves on the AChE [27]. Yang et al. reported potent AChE inhibitors isolated from seeds of *Peganum harmala* [28]. In our study, treatment with the herbal plant *P. chinense* ethanol extract led to a dramatic increase in AChE activity inhibition. In contrast, no significant effect was observed on amyloid-β aggregation, which is another key characteristic of Alzheimer’s disease [29]. The two standard components of *P. chinense* also increased the inhibition of AChE in a dose-dependent manner.

Oxidative stress plays a critical role in the pathogenesis of Alzheimer’s disease [30]. A large number of herbal medicines or their phytochemicals have been reported to have strong antioxidant activities by scavenging free radicals, thus acting as anti-Alzheimer’s disease drugs [31,32]. We conducted an ABTS radical scavenging activity assay to assess the antioxidant effects of the *P. chinense* extract and of its two chemical components. As expected, the *P. chinense* extract dose-dependently increased the ABTS radical scavenging activity. However, scavenging activity was detected for phellodendrine, but not for berberine. To date, several articles have reported the free radical scavenging activity of berberine extracted from various herbal plants [33,34,35]. In comparison with those reports, we used lower concentrations of berberine (≤2 μM = 0.67 μg/mL) because of its cytotoxicity against neuronal cells at ≥2 μM (data not shown). According to Shirwaikar et al., the ABTS scavenging activity of berberine occurred in a dose-dependent manner, with maximum activity at 512 μg/mL and minimum activity at 2 μg/mL (IC_50_ = 38.7 μg/mL) [36]. Thus, phellodendrine, rather than berberine, may be considered as the active compound from *P. chinense* with anti-Alzheimer’s activity, although its amount in the *P. chinense* extract was lower than that of berberine.

The clinical symptoms of Alzheimer’s disease are thought to be associated with the degree of neuronal loss in the hippocampus and cerebral neocortex [36]. Therefore, we further confirmed the anti-Alzheimer’s activity of *P. chinense* by analyzing its neuroprotective effect. Neuronal cell damage was induced by exposing HT22 hippocampal cells to H_2_O_2_ and measuring the release of LDH. LDH is an enzyme that catalyzes the conversion of lactate to pyruvate. It is found in living cells and released from damaged tissues or cells. Treatment with H_2_O_2_, which is an inducer of oxidative stress in neuronal cells [37,38], significantly stimulated LDH release, while the concurrent administration of H_2_O_2_ and the *P. chinense* extract significantly reduced the H_2_O_2_-mediated LDH levels. These results suggest that *P. chinense* has the ability to protect against neuronal cell death.

In summary, our investigation successfully established an HPLC analytical method for the quantification of the two standard components of *P. chinense* and will be helpful for the quality control of *P. chinense.* Furthermore, our findings suggest the potential of phellodendrine as an active component of *P. chinense* for targeting Alzheimer’s disease. Additional experiments will be required to verify the anti-Alzheimer’s activities of *P. chinense* and phellodendrine and to understand their regulatory mechanisms using in vitro and in vivo models of Alzheimer’s disease.

## 4. Materials and Methods

### 4.1. Plant Material

The bark of *Phellodendron chinense* used in this study was purchased from the Kwangmyungdang herbal market (Ulsan, Korea) and identified by Dr. Goya Choi, K-herb Research Center, Korea Institute of Oriental Medicine, Daejeon, Korea. A voucher specimen (Phch5-LE) has been deposited at the Herbal Medicine Research Division, Korea Institute of Oriental Medicine.

### 4.2. Chemicals and Reagents

The standard components, phellodendrine and berberine, were purchased from Biopurify Phytochemicals Ltd. (Chengdu, China) and ChemFaces Biochemical Co., Ltd. (Wuhan, China), respectively. The chemical structures of the standard components are shown in Figure 1. The purity of these standard components was ≥98.0%, as assessed using HPLC. The HPLC-grade solvents, acetonitrile and water were purchased from J. T. Baker Chemical Co. (Phillipsburg, NJ, USA), and the analytical-grade reagent trifluoroacetic acid (TFA) was purchased from Sigma-Aldrich (St. Louis, MO, USA).

### 4.3. Apparatus and Chromatographic Conditions

Quantitative analysis was conducted using a Waters Alliance e2695 system (Waters Corp., Milford, MA, USA) equipped with a pump, degasser, column oven, autosampler, and photodiode array detector (#2998; Waters Corp.). The data were acquired and processed using Empower software (version 3; Waters Corp.). The chromatographic separation of the two standard components was carried out at room temperature using Luna C_18_ analytical columns (250 mm × 4.6 mm, 5 μm) supplied by Phenomenex (Torrance, CA, USA), with a gradient solvent system of acetonitrile and water. The UV wavelengths used for detection were 200 nm for phellodendrine and 226 nm for berberine. The flow rate was 1.0 mL/min and the injection volume was 10 μL.

### 4.4. Preparation of Standard Solutions

The two components were weighed accurately, dissolved in methanol at 1.0 mg/mL, and stored at <4 °C. Each stock solution was used for quantitative analysis after serial dilution with methanol.

### 4.5. Preparation of Sample Solutions

The dried bark of *P. chinense* (45 g) was extracted twice with 70% ethanol (270 mL) by refluxing for 2 h. The extracted solution was filtered through a filter paper (5 μm) and evaporated using a rotary evaporator (EYELA N-1000; Rikakikai Co., Tokyo, Japan) under vacuum to give a powdered 70% ethanol extract (9.03 g). The yield of *P. chinense* extract was 20%. For quantitative analysis, the powdered 70% ethanol extract of *P. chinense* was weighed accurately and dissolved in methanol at a concentration of 2.5 mg/mL. The sample solution was filtered through a syringe filter (0.45 μm) before HPLC analysis.

### 4.6. Calibration Curves and Limits of Detection and Quantification

The calibration curves of the components were obtained by assessment of the peak areas of the standard solutions at five different concentrations. The tested concentration ranges were 12.5–200 μg/mL for phellodendrine and 62.5–1000 μg/mL for berberine. The LOD and LOQ for the two standard components were calculated using the slope of the calibration curve and the standard deviation (SD) of the intercept, as follows:

LOD = 3.3 × (SD of the response/slope of the calibration curve) LOQ = 10 × (SD of the response/slope of the calibration curve).(1)

### 4.7. In Vitro AChE Activity Assay

In vitro AChE activity was assessed according to a protocol based on Ellman’s colorimetric method [39], with modifications using a Acetylcholinesterase Assay Kit (Abcam, Cambridge, UK). Stock solution of 70% ethanol extract from *P. chinense* was dissolved in DMSO at a concentration of 100 mg/mL. Preparation of 70% ethanol extract from *P. chinense* was addressed above in Section 4.5. Assay samples were prepared by diluting in 0.1 M sodium phosphate buffer (pH 8.0). In detail, the 70% ethanol extract of *P. chinense* at a concentration of 100 mg/mL was diluted in 0.1 M sodium phosphate buffer (pH 8.0) to a final concentration of 100, 50, 25, and 12.5 μg/mL of serial dilutions. The two standard compounds phellodendrine (100 mM in DMSO) and berberine (1 mM in DMSO) were dissolved in 0.1 M sodium phosphate buffer (pH 8.0) to a final concentration of 100, 50, 25 and 12.5 μM, and 1, 0.5, 0.25 and 0.125 μM of serial dilutions, respectively. The AChE stock solution was prepared by dissolving in 0.1% bovine serum albumin/H_2_O at 25 U/mL and dissolved in 0.1 M sodium phosphate buffer (pH 7.3, assay buffer), to a final concentration of 35.2 mU/mL, before the assay. The substrates acetylthiocholine iodide and 5,5′-dithiobis-2-nitrobenzoic acid (DTNB) were dissolved in H_2_O and assay buffer, respectively, at 10 mM, and both solutions were diluted 20-fold in assay buffer to a final concentration of 0.5 mM. In detail, 0.6 mL of H_2_O and assay buffer were added in acetylthiocholine iodide and DTNB, respectively, to make 10 mM stock solution. For assays, 0.25 mL of each 10 mM acetylthiocholine iodide and DTNB was mixed in 4.75 mL of assay buffer to a final concentration of 0.5 mM and used as the reaction mixture. For the enzymatic reaction in 96-well plates, 50 μL of the sample solution and 50 μL of the reaction mixture were mixed and preincubated for 10 min at room temperature. The 10 μL of AChE solution was then added to the initial mixture to start the reaction, which was carried out for 1 h at room temperature. The absorbance was measured at 412 nm using an Epoch microplate spectrophotometer (Bio-Tek Instruments, Winooski, VT, USA). The inhibition of the activity of AChE was calculated by comparing the rate of reaction of the sample to that of the blank. All experiments were performed in triplicate, and the percentage of inhibition against the AChE activity was calculated according to the following equation:

(2) $$\mathrm{AChE}\;\mathrm{activity}\;\mathrm{inhibition}\;\left(\%\right)=1-\frac{S\;–\;S’}{C\;–\;C’}\;\times \;100$$

S: samples (sample solution, assay buffer with DTNB, substrate, and enzyme)
S’: samples (sample solution, assay buffer with DTNB and substrate, without enzyme)
C: control (0.1 M sodium phosphate buffer (pH 8.0), assay buffer with DTNB, substrate, and enzyme)
C’: control (0.1 M sodium phosphate buffer (pH 8.0), assay buffer with DTNB and substrate, without enzyme)

### 4.8. In Vitro Amyloid-β Aggregation Assay

Amyloid-β (1–42) aggregation was measured using the SensoLyte^®^ Thioflavin T β-Amyloid aggregation kit (AnaSpec, Fremont, CA, USA), according to the manufacturer’s instructions. The assay is based on the property of the Thioflavin T dye, the fluorescence of which increases when it is bound to aggregates of amyloid-β (1–42) peptides. Briefly, Thioflavin T was dissolved in assay buffer (50 mM Tris/150 mM NaCl (pH = 7.2), 20 mM HEPES/150 mM NaCl (pH = 7.2), 10 mM phosphate/150 mM NaCl) and used at a concentration of 100 μM. Samples were dissolved in assay buffer and used at a final concentration of 100 μg/mL. To measure the inhibition of amyloid-β (1–42) aggregation in 96-well black microplates, 5 μL of the sample and 85 μL of amyloid-β (1–42) were mixed, followed by the addition of 10 μL of Thioflavin T. Thioflavin T fluorescence was measured at intervals of 20 min for 2 h, with an excitation wavelength (λ_ex_) of 440 nm and an emission wavelength (λ_em_) of 485 nm using a SpectraMax i3 Multi-Mode Detection Platform (Molecular Devices, Sunnyvale, CA, USA). This gave seven readings for each well sample. All fluorescence readings are expressed in relative fluorescence units. Morin was used as a positive control. Experiments were performed in triplicate and averaged, and the percentage of inhibition of amyloid-β aggregation was calculated according to the following equation:

(3) $$\mathrm{Amyloid}-\beta \;\mathrm{aggregation}\;\mathrm{inhibition}\;\left(\%\right)=1-\frac{S-S’}{C-C’}\;\times \;100$$

S: samples (sample solution, assay buffer with ThT, amyloid-β solution)
S’: samples (sample solution, assay buffer with ThT, without amyloid-β solution)
C: control (assay buffer with ThT, amyloid-β solution)
C’: control (assay buffer with ThT, without amyloid-β solution)

### 4.9. ABTS Radical Scavenging Activity

The 2,2′-Azino-bis(3-ethylbenzothiazoline-6-sulphonic acid) (ABTS) radical cations were produced by reacting a 7 mM ABTS solution with 2.45 mM potassium persulfate in the dark at room temperature for 16 h. The absorbance of the reactant was later adjusted to 0.7 at a wavelength of 734 nm. Aliquots of *P. chinense* extract solution (100 μL) at various concentrations were mixed with 100 μL of ABTS^•+^ solution. The reaction mixture was incubated for 5 min in the dark at room temperature. The absorbance of the resulting solution was measured at 734 nm using an Epoch microplate spectrophotometer (Bio-Tek Instruments). The radical scavenging capacity of the *P. chinense* extract-treated samples was calculated using the following equation:

(4) $$\mathrm{Scavenging}\;\mathrm{activity}\;\left(\%\right)=\frac{1-\mathrm{Absorbance}\;\mathrm{of}\;P.\;chinense\;\mathrm{extract}-\mathrm{treated}\;\mathrm{sample}}{\mathrm{Absorbance}\;\mathrm{of}\;\mathrm{untreated}\;\mathrm{sample}}\;\times \;100$$

### 4.10. CCK Assay

The HT22 cells were maintained in Dulbecco’s modified Eagle’s medium (Hyclone/Thermo, Rockford, IL, USA) supplemented with 10% fetal bovine serum (Hyclone/Thermo) and penicillin/streptomycin in 5% CO_2_ at 37 °C.

To determine the nontoxic concentration of the *P. chinense* extract in HT22 cells, a CCK assay was performed using a CCK-8 assay kit (Dojindo, Kumamoto, Japan). Cells were plated in 96-well microplates at a density of 5 × 10^3^ cells/well and treated with various concentrations (0, 12.5, 25, 50 and 100 μg/mL) of the *P. chinense* extract for 24 h. The CCK-8 solution was added and the cells were incubated for 4 h. Absorbance was read at 450 nm on an Epoch microplate spectrophotometer (Bio-Tek Instruments). Cell viability was calculated using the following equation:

(5) $$\mathrm{Cell}\;\mathrm{viability}\;\left(\%\right)=\frac{\mathrm{Mean}\;\mathrm{OD}\;\mathrm{in}\;P.\;chinense\;\mathrm{extract}-\mathrm{treated}\;\mathrm{cells}}{\mathrm{Mean}\;\mathrm{OD}\;\mathrm{in}\;\mathrm{untreated}\;\mathrm{cells}}\times 100$$

### 4.11. LDH Release Assay

To determine the neuroprotective effect of the *P. chinense* extract, the release of LDH was measured in H_2_O_2_-damaged HT22 cells using the CytoTox 96 nonradioactive cytotoxicity assay kit (Promega, Madison, WI, USA). Cells were lysed to induce maximal LDH release, and supernatants were collected to measure experimental LDH release. Cell lysates or supernatants were reacted with substrate mixture at room temperature for 30 min in the dark. After adding the stop solution, absorbance at 490 nm was measured on an Epoch microplate spectrophotometer (Bio-Tek Instruments). The cytotoxicity of the *P. chinense* extract was calculated using the following formula:

(6) $$\mathrm{Cytotoxicity}\;\left(\%\right)=\frac{\mathrm{Experimental}\;\mathrm{LDH}\;\mathrm{release}\;}{\mathrm{Maximum}\;\mathrm{LDH}\;\mathrm{release}}\;\times \;100$$

### 4.12. Statistical Analysis

The data are expressed as the mean ± SEM. Data were analyzed using one-way analysis of variance and Dunnett’s multiple comparisons test. *p* < 0.05 was considered significant.

## Figures and Tables

**Table 1:** Condition of mobile phase for HPLC analysis.

Time (min)	Flow Rate (mL/min)	Mobile Phase	
		0.1% Trifluoroacetic Acidin Water (%)	Acetonitrile (%)
0	1.0	100	0
40	1.0	55	45
45	1.0	0	100
52	1.0	0	100

**Table 2:** Linear range, regression equation, correlation coefficients, limits of detection (LODs), and limits of quantitation (LOQs) for compounds.

Compound	Linear Range (μg/mL)	Regression Equation (*y* = a*x* + b) ^(a)^		Correlation Coefficient (*r*^2^)	LOD ^(b)^ (μg/mL)	LOQ ^(c)^ (μg/mL)
		Slope ^(a)^	Intercept ^(b)^			
Phellodendrine	12.5–200	52,064	222,327	0.9991	0.301	0.911
Berberine	62.5–1000	40,183	434,513	0.9998	1.555	4.712

^(**a**)^ *y* = a*x* + b, *y* means peak area and *x* means concentration (μg /mL); ^(b)^ LOD: 3.3 × (standard deviation (SD) of the response/slope of the calibration curve); ^(c)^ LOQ: 10 × (SD of the response/slope of the calibration curve).

**Table 3:** The content of marker compounds in *P. chinense*.

Compound	Content (mg/g)
Phellodendrine	22.255 ± 0.123
Berberine	269.651 ± 1.257

**Table 4:** Inhibitory activity of *P. chinense* on in vitro acetylcholinesterase (AChE) activity and amyloid-β aggregation (at 100 μg/mL).

Inhibition of AChE Activity (%)	Inhibition of Amyloid-β Aggregation (%)
98.92 ± 0	−7.54 ± 5.74
